# Anisotropic in-plane thermal conductivity observed in few-layer black phosphorus

**DOI:** 10.1038/ncomms9572

**Published:** 2015-10-16

**Authors:** Zhe Luo, Jesse Maassen, Yexin Deng, Yuchen Du, Richard P. Garrelts, Mark S Lundstrom, Peide D. Ye, Xianfan Xu

**Affiliations:** 1 School of Mechanical Engineering, Purdue University, West Lafayette, Indiana 47907, USA; 2 Birck Nanotechnology Center, Purdue University, West Lafayette, Indiana 47907, USA; 3 School of Electrical and Computer Engineering, Purdue University, West Lafayette, Indiana 47907, USA

## Abstract

Black phosphorus has been revisited recently as a new two-dimensional material showing potential applications in electronics and optoelectronics. Here we report the anisotropic in-plane thermal conductivity of suspended few-layer black phosphorus measured by micro-Raman spectroscopy. The armchair and zigzag thermal conductivities are ∼20 and ∼40 W m^−1^ K^−1^ for black phosphorus films thicker than 15 nm, respectively, and decrease to ∼10 and ∼20 W m^−1^ K^−1^ as the film thickness is reduced, exhibiting significant anisotropy. The thermal conductivity anisotropic ratio is found to be ∼2 for thick black phosphorus films and drops to ∼1.5 for the thinnest 9.5-nm-thick film. Theoretical modelling reveals that the observed anisotropy is primarily related to the anisotropic phonon dispersion, whereas the intrinsic phonon scattering rates are found to be similar along the armchair and zigzag directions. Surface scattering in the black phosphorus films is shown to strongly suppress the contribution of long mean-free-path acoustic phonons.

## Abstract (web summary)

The two-dimensional material black phosphorus could find uses in energy applications. Here, the authors study the difference in in-plane thermal conductivity along the armchair and zigzag directions in suspended few-layer black phosphorus, and show the dependence of this anisotropy on sample thickness.

## Graphical abstract

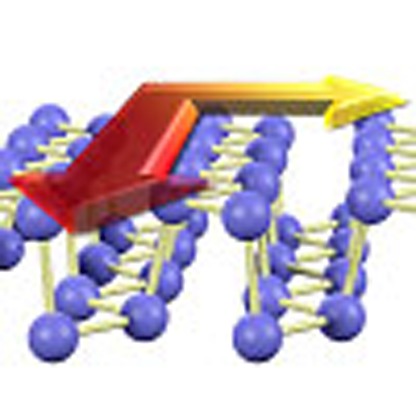

The successful isolation of graphene^1^,^2^ has inspired rapidly growing research efforts on two-dimensional (2D) materials in the last decade, among which the extensively studied ones include transition metal dichalcogenides^3^,^4^,^5^ and hexagonal boron nitride (hBN)^6^,^7^. The 2D materials attract an enormous amount of interest, owing to their extraordinary electronic, optical and mechanical properties comparing with bulk counterparts. Recently, black phosphorus (BP) was revisited as a new 2D material with high hole mobility and moderate on/off ratio demonstrated on few-layer BP field-effect transistors^8^,^9^,^10^,^11^,^12^,^13^. Unlike semi-metallic graphene with zero band gap^2^ and MoS_2_ with a direct band gap of ∼1.8 eV only in its monolayer form^5^, BP exhibits a thickness-dependent direct band gap varying from ∼0.3 eV (bulk) to > 1.4 eV (monolayer)^9^,^14^,^15^,^16^. Such tunable band gap benefits few-layer BP in optoelectronic applications such as phototransistors, p–n diodes and solar cells^10^,^17^,^18^,^19^,^20^. In addition, BP shows intriguing anisotropic properties, owing to the puckered nature of its in-plane lattice. Several works have explored anisotropic transport in BP, which enables BP for potential applications in novel electronic and optoelectronic devices where the anisotropic properties might be used^10^,^21^,^22^,^23^.

Although electronic and photovoltaic properties have been extensively investigated, thermal transport studies of BP, especially experimental ones, are still lacking. Recently, the thermoelectric power of bulk BP has been reported, indicating that BP could be used as an efficient thermoelectric material at around 380 K^24^. Some recent first-principles studies also raised interest of BP in thermoelectric applications, claiming that because of the anisotropic lattice structure, the ‘armchair' direction possesses high electrical conductivity and low lattice thermal conductivity, which is desirable for thermoelectrics^21^,^25^,^26^,^27^,^28^. On the contrary, such ‘orthogonal' electronic and phononic transport of BP may not be favourable in typical field-effect transistor and photovoltaic devices, as low thermal conductivity in the channel direction can lead to thermal management issues. However, to the best of our knowledge, the only thermal conductivity measurement of BP was conducted in 1965 by Slack^29^ on a bulk poly-crystalline BP sample, which neither addressed the anisotropic thermal properties of BP nor the thermal transport particularly in few-layer BP.

Here we report the anisotropic in-plane thermal conductivity of suspended few-layer BP measured by micro-Raman spectroscopy, which has been used for measuring thermal conductivity of 2D materials such as graphene^30^,^31^, MoS_2_ (refs 32, 33) and hBN^34^. Under laser illumination, the local temperature rise leads to softened atomic bonds and anharmonic phonon coupling as found in graphene^35^, inducing a change in the optical phonon frequency and causing the red shift of the corresponding Raman peaks. Such laser-induced temperature-dependent Raman scattering is used as an optical thermometer, whereas the focused laser itself acts as a steady-state heat source. In this work, we produced few-layer BP films of 9.5–29.6 nm thickness via the ‘Scotch tape' method and suspended them on 3-μm-wide slits fabricated on free-standing silicon nitride (SiN) substrate films to measure the in-plane thermal conductivity of BP using the micro-Raman technique. The measured thermal conductivity ranges from ∼10 to ∼20 W m^−1^ K^−1^ along the armchair direction and ∼20 to ∼40 W m^−1^ K^−1^ along the zigzag direction, showing strong thickness dependence and significant anisotropy in the *x*–*y* plane. Theoretical calculations of heat transport properties of few-layer BP, based on a first-principles phonon dispersion and a phenomenological treatment of scattering, demonstrate that the anisotropic phonon dispersion along the two directions is responsible for the observed anisotropy, as the scattering rates are found to be nearly isotropic. The thickness dependence of the thermal conductivity is attributed to the strong surface scattering of acoustic phonons, especially phonons with long mean-free-path (MFP).

## Results

### Sample preparation and polarized Raman characterization

**Figure 1:**
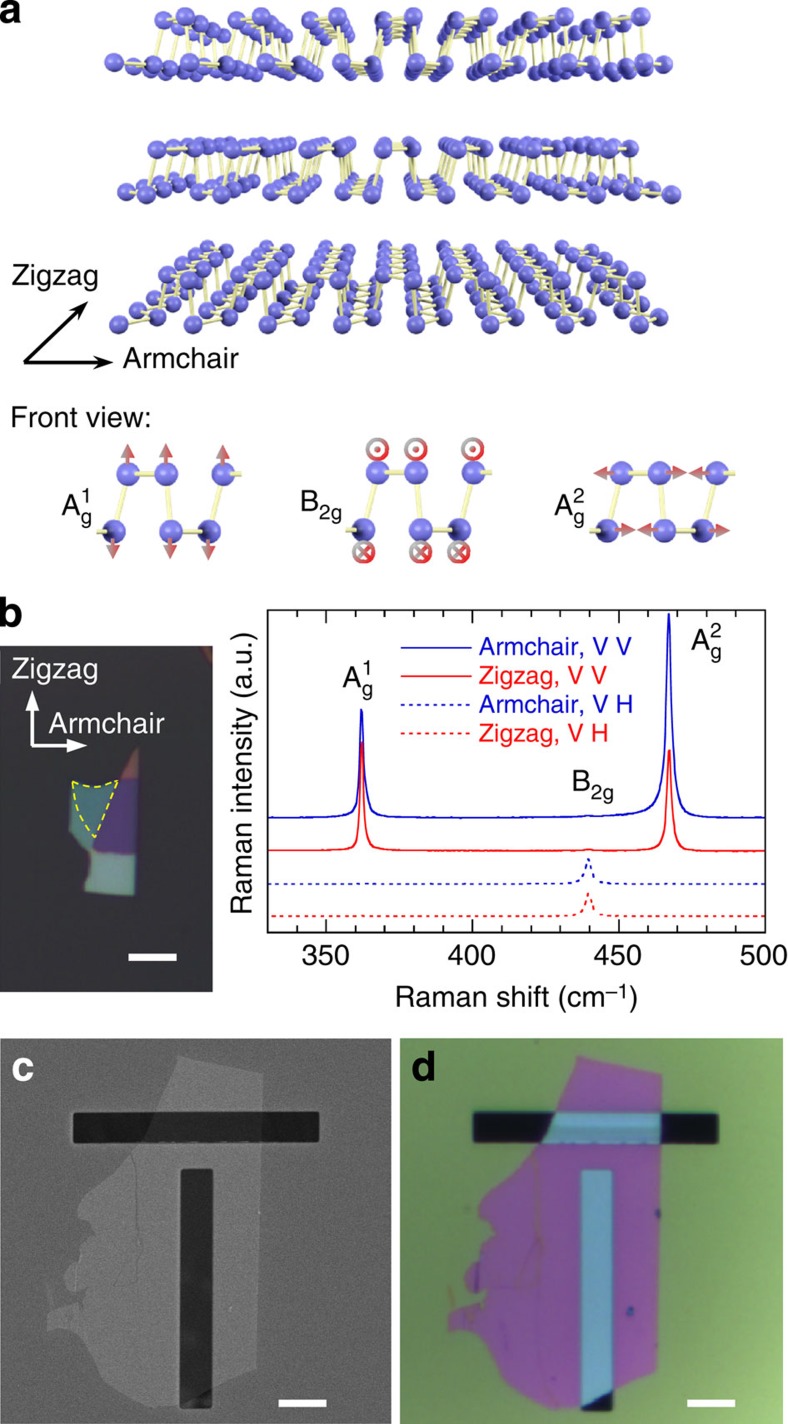
Characterization of black phosphorus flakes. (**a**) Lattice structure of BP and atomic vibrational patterns of A_g_^1^, B_2g_ and A_g_^2^ phonon modes. (**b**) Polarized Raman spectra (right) collected from a BP flake on SiO_2_/Si substrate (left) showing the ability of polarized Raman technique to distinguish the armchair and zigzag axes. (**c**) Scanning electron microscopy image and (**d**) optical image of the 16.1-nm-thick BP flake suspended on slits. Scale bars, 5 μm (all).

Bulk BP consists of puckered honeycomb atomic sheets bonded by van der Waals force and therefore can be mechanically exfoliated into atomically thin layers. Figure 1a illustrates its layered lattice structure. BP flakes were tape exfoliated from bulk crystals and then released onto transparent polymer films for optical examination under a microscope. The large, visually uniform and transparent ones were selected as candidates. Before the flakes were transferred, the two principal lattice axes, which are generally referred to as the ‘armchair' and ‘zigzag' axes, were determined using polarized Raman spectroscopy. To observe the polarized Raman scattering, a linear polarizer was placed at the spectrometer entrance. With the detection polarization perpendicular (referred to as ‘VH configuration' where ‘V' stands for vertical laser polarization and ‘H' for horizontal detection polarization) or parallel (VV configuration) to the incident laser polarization, the optical phonon modes of different symmetries can be selected or eliminated when lattice principal axes are aligned with the laser polarization. In the case of BP, the A_g_ modes and the B_2g_ mode can be filtered out in VH and VV configurations, respectively, when either the armchair or zigzag axis is aligned with the laser polarization, as shown in Fig. 1b. In this way we were able to identify the armchair or zigzag axis by, for example, observing the B_2g_ mode Raman intensity in the VV configuration, while rotating the BP flake. To further distinguish these two axes, we looked into the A_g_^2^/A_g_^1^ Raman intensity ratio in the VV configuration. The armchair-oriented atomic vibrations of A_g_^2^ phonons lead to maximized A_g_^2^ Raman intensity when laser polarization is along the armchair direction, whereas the A_g_^1^ Raman intensity remains unchanged because the A_g_^1^ phonon vibrations are out-of-plane^36^ (Fig. 1a). Therefore, the A_g_^2^/A_g_^1^ intensity ratio becomes larger (∼2) with armchair-polarized laser excitation and smaller (∼1) with zigzag-polarized laser excitation (Fig. 1b), which serves as Raman signatures of armchair and zigzag lattice axes. The polarized Raman method is simple yet effective and accurate in determining the lattice orientation of BP crystals. More details of the polarized Raman measurements can be found in Supplementary Fig. 1 and Supplementary Note 1.

Upon determination of the lattice orientation, the candidate BP flakes were aligned (with angular uncertainty less than ± 3°) and transferred to 3-μm-wide slits fabricated on 200-nm-thick free-standing SiN membranes, details of which can be found in Methods and Supplementary Fig. 2. The rectangular geometry, along with a nearly one-dimensional laser heat source in the centre, which will be described later, guarantees that the heat conduction is most sensitive to the thermal conductivity perpendicular to the slit. Two slits were patterned to be mutually perpendicular to form a ‘T' shape, so that the armchair and zigzag thermal transport can be separately investigated on the same flake. Scanning electron microscope (SEM) images and optical images were taken for surface characterization purpose, and multiple atomic force microscope (AFM) scans were performed to obtain the thickness of the flake and to confirm the thickness uniformity. Figure 1c,d are, respectively, SEM and optical images of a successfully transferred 16.1-nm-thick flake on T-shaped slits. Polarized Raman spectra were also collected on the suspended areas to ensure that the lattice was correctly aligned with the slit. For all subsequent Raman measurements, unpolarized detection was used instead of polarized detection, to achieve maximum signal collection efficiency and reduce the measurement uncertainty.

### Thermal measurements by micro-Raman spectroscopy

**Figure 2:**
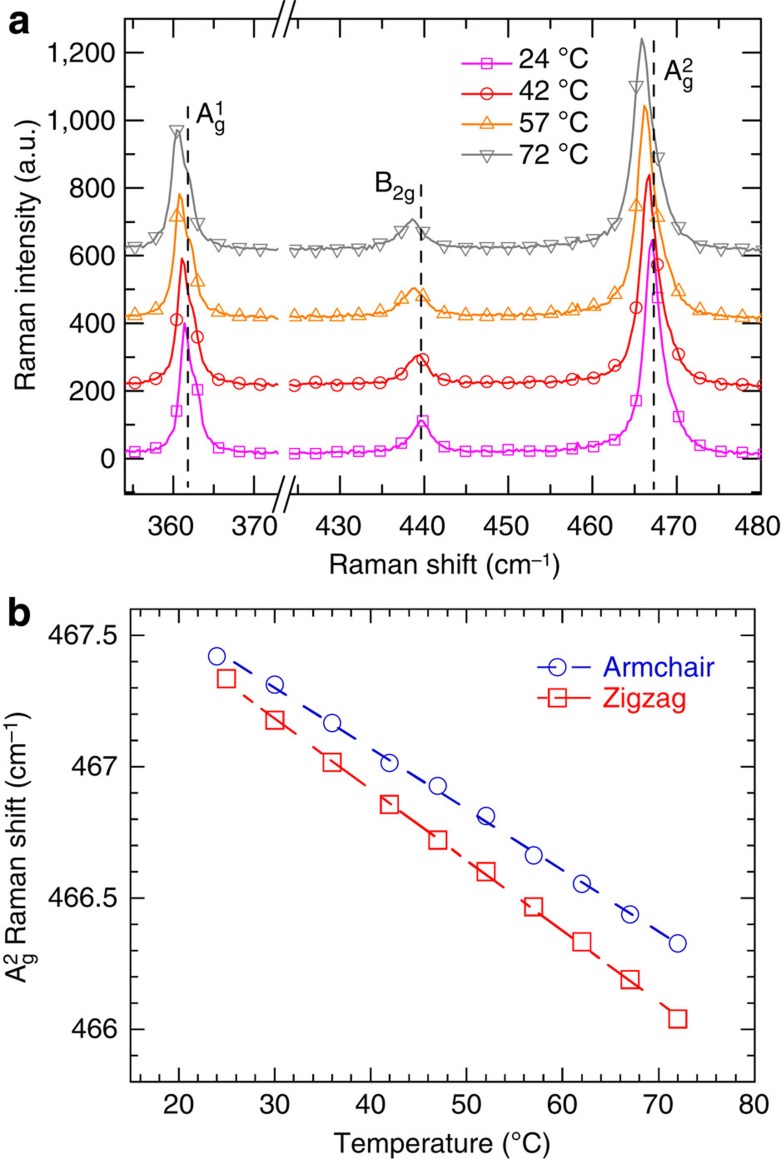
Raman thermometer calibration results of the 9.5-nm-thick BP film. (**a**) Four sample Raman spectra taken at 24, 42, 57 and 72 °C with armchair-polarized laser. The dashed lines correspond to the peak positions at 24 °C. (**b**) The A_g_^2^ Raman shift as a function of temperature for both armchair- and zigzag-polarized laser. The dashed lines show linear fit results.

The Raman optical thermometer, that is, the temperature-dependence of Raman scattering, was calibrated using a Raman spectrometer and a heating stage purged with nitrogen. Throughout the calibration, the excitation laser power used was <150 μW, to minimize excessive heating effects. Taking into consideration the anisotropy, separate calibrations were performed with both armchair- and zigzag-polarized laser excitations. Figure 2a shows several Raman spectra at different temperatures collected from the 9.5-nm-thick suspended BP film under armchair-polarized excitation, in which the Raman peaks shift towards lower frequency on heating. In the small-temperature-rise regime, the Raman mode frequency can be expressed as *ω*=*ω*_0_+*χθ* with higher-order terms neglected, where *ω*_0_ is the frequency at room temperature, *θ* is the temperature rise and *χ* is the temperature coefficient^33^. For the 9.5-nm-thick film, the extracted temperature coefficients are *χ*_armchair,Ag1_=−0.01895, cm^−1^ K^−1^, *χ*_armchair,B2g_=−0.02434, cm^−1^ K^−1^, *χ*_armchair,Ag2_=−0.02316, cm^−1^ K^−1^, *χ*_zigzag,Ag1_=−0.02175, cm^−1^ K^−1^, *χ*_zigzag,B2g_=−0.02877, cm^−1^ K^−1^ and *χ*_zigzag,Ag2_=−0.02700, cm^−1^ K^−1^, which are in agreement with previously reported values^37^. It is seen that the zigzag-polarized excitation yielded temperature coefficients of larger absolute values (see Fig. 2b for A_g_^2^ mode as an example), which might be caused by anisotropic thermal expansion during the heating process. Among the three Raman modes, the A_g_^2^ mode was found to be most sensitive to temperature change, as well as showing highest Raman intensity; therefore, it was chosen as the thermometer. Raman shift is also sensitive to strain and stress, aside from temperature change. The evaluation of such effects is provided in Supplementary Note 2.

**Figure 3:**
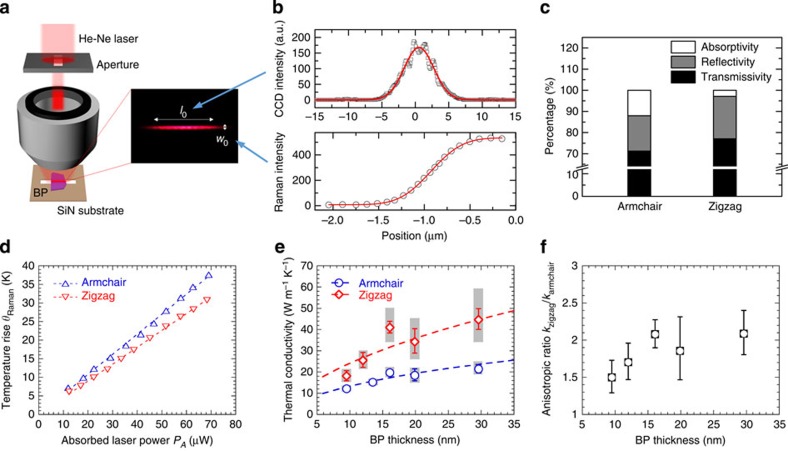
Thermal conductivity measurements of BP using micro-Raman technique. (**a**) Illustration of the experimental setup and an optical image of the produced laser focal line. (**b**) The lengthwise profile and the knife-edge-measured widthwise integrated profile of the laser focal line. The solid lines are Gaussian function and error function curve fits, respectively. (**c**) The optical absorptivity *A*, reflectivity *R* and transmissivity *T* of the 9.5-nm-thick suspended BP film on armchair- and zigzag-polarized laser incidence. (**d**) Laser-power-dependent temperature rise (*θ*_Raman_) of the 16.1-nm-thick BP film determined by the micro-Raman spectroscopy along armchair and zigzag transport directions. The dashed lines are linear fits. (**e**) Extracted armchair and zigzag in-plane thermal conductivities (*k*_armchair_ and *k*_zigzag_) of multiple BP films. Dashed lines are results of theoretical modelling. The grey error bars account for the uncertainty of SiN substrate thermal conductivity *k*_SiN_, whereas the blue/red error bars do not. (**f**) The anisotropic ratio *k*_zigzag_/*k*_armchair_ at different BP thicknesses. The ratio at 12-nm thickness is calculated using linearly interpolated armchair thermal conductivity from adjacent thicknesses.

Micro-Raman thermal conductivity experiments were conducted at room temperature in nitrogen atmosphere. Figure 3a illustrates the experimental setup. To best achieve the desired one-dimensional heat transfer, a 75-μm-wide rectangular aperture was placed in front of the objective lens to produce a laser focal line instead of a circular spot. In the direction where the aperture cuts the laser beam, the partially filled objective lens aperture produces a larger width at the focal point, yielding a stretched line-shaped focal spot. The Gaussian width *w*_0_ and length *l*_0_ (both defined as the radius where the intensity drops to 1/*e*^2^) of the laser focal line is characterized to be 0.39 and 3.1 μm, respectively (Fig. 3b). The laser focal line was aligned to the centreline of the slit, creating a one-dimensional heat source at the centre of the suspended BP film. Consequently, the dominant heat flow occurred perpendicular to the slit, enabling the isolation of heat conduction along the armchair or zigzag direction.

At various incident laser powers, a series of Raman spectra were collected and converted to the local temperature rise at the laser focal line using the previously obtained temperature coefficient. Figure 3d plots the Raman-measured temperature rise *θ*_Raman_ versus the absorbed laser power *P*_*A*_ for the 16.1-nm-thick suspended BP film, in which the data show a linear correlation similar to other micro-Raman experiments^30^,^31^,^32^,^33^,^34^. The absorbed laser power was determined by *P*_*A*_=*AP*=(1−*R*−*T*)*P*, where *P* is the incident laser power, *A* is the absorptivity, *R* is the reflectivity and *T* is the transmissivity. The reflectivity of BP films is measured using a beam splitter in the incident laser path, which deviates the reflected light to a separate path where its intensity is measured; by comparing the reflected light intensity of the BP films and a silver-coated mirror as a reference, the reflectivity of BP films can be calculated. The transmissivity of the BP films is measured under the slit, by dividing the transmitted laser intensity on the BP-covered slit by that at the adjacent empty slit. It is noteworthy that *A*, *R* and *T* are all anisotropic quantities (Fig. 3c), owing to anisotropic optical conductivity, and our measured absorptivity of the 9.5-nm-thick suspended film (∼12.0% for armchair polarization and ∼2.9% for zigzag polarization) agrees well with the theoretical predictions^22^. Owing to the much higher absorption of armchair-polarized light in BP films, all the thermal conductivity measurements were carried out with an armchair-polarized laser beam, to reduce the uncertainty of *P*_*A*_. The uncertainty of the absorptivity of the BP films we measured ranges from 0.2% to 0.7%.

### Extracting the in-plane thermal conductivity of BP

A three-dimensional anisotropic heat conduction equation


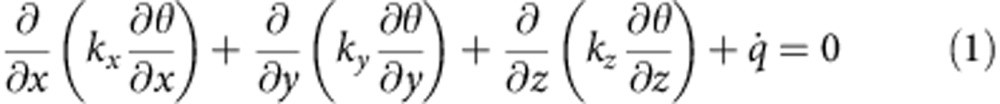


was solved using the finite volume method, with the heat source term for stretched laser focal line given as


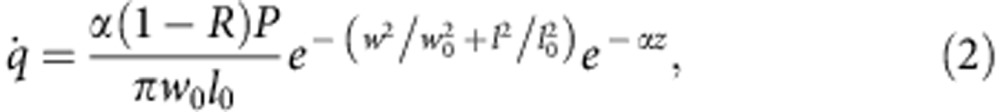


where *α* is the absorption coefficient, and *w* and *l* represent the coordinates corresponding to the width and length directions of the focal line, respectively. The temperature rise within the laser focal line is expressed in Gaussian-weighted-average form


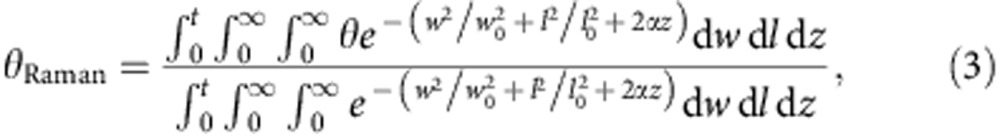


where *t* is the BP film thickness and the factor 2 comes from the absorption of Raman scattered photons within the film when they are travelling backwards to the surface. It is shown that theoretically *θ*_Raman_ varies linearly with absorbed laser power *P*_*A*_ for thin films^38^. By comparing the experimentally measured slopes d*θ*_Raman_/d*P*_*A*_ with the calculated slopes, one can extract both the armchair and zigzag thermal conductivity (*k*_armchair_ and *k*_zigzag_) of the suspended BP film by iterative calculations between the two directions (Supplementary Note 3), as even though the temperature is predominately affected by the thermal conductivity along the direction perpendicular to the slit, it is still weakly affected by the thermal conductivity along the other direction. It is also worth noting that, although the in-plane thermal transport dominates in the suspended region, the cross-plane conduction may need to be accounted for in the supported region, as it directly affects the heat sink efficiency. Therefore, we evaluated the cross-plane heat conduction in the supported region and performed separate micro-Raman and time-domain thermoreflectance measurements of the thermal conductivity of the SiN substrate, details of which are included in Supplementary Fig. 3 and Supplementary Note 4.

## Discussion

The measured anisotropic in-plane thermal conductivity values of few-layer BP are summarized in Fig. 3e. The electronic contribution to the total thermal conductivity is estimated to be a small fraction given typical carrier concentrations in few-layer BP (Supplementary Note 5); therefore, the data presented here can be largely attributed to the lattice. The measured *k*_zigzag_ is generally 50%–100% higher than *k*_armchair_, showing strong anisotropic feature^21^. It arises mostly from the anisotropic phonon dispersion along Γ–X (armchair) and Γ–A (zigzag) directions as discussed further below. In Fig. 3e, *k*_zigzag_ starts from ∼40 W m^−1^ K^−1^ for thick films over 15 nm, then sharply decreases to ∼20 W m^−1^ K^−1^; similar trend can also be found for *k*_armchair_, decreasing from ∼20 to ∼10 W m^−1^ K^−1^. This strong thickness dependence originates from significant surface scattering of long-MFP phonons; similar results were observed in few-quintuple-layer Bi_2_Te_3_ films^39^, where surface scattering was found to heavily affect electron and phonon transport. The anisotropic trend of larger thermal conductivity along zigzag compared with armchair direction is consistent with previous theoretical calculations^25^,^26^,^40^. The values of 110 W m^−1^ K^−1^ (zigzag) and 36 W m^−1^ K^−1^ (armchair) predicted through first-principles modelling by Jain and McGaughey^26^ are larger compared with those reported in this work. Other *ab initio* calculations^25^,^40^ provided thermal conductivities closer to our experimental values, but these smaller values were shown to result from approximations in the theoretical approach^26^. The predicted thermal conductivities were obtained assuming perfect monolayer BP; however, in the presence of surface roughness or adsorbates, greater phonon scattering can occur leading to smaller thermal conductivities, which is the main source of discrepancy between the previous predictions and the measured values reported here. The measured thermal conductivity of few-layer BP is comparable with the reported value of bulk BP (12.1 W m^−1^ K^−1^)^29^; however, the bulk BP was poly-crystalline so that this thermal conductivity value was merely an averaged value along three lattice axes, among which the cross-plane axis exhibits lower thermal conductivity, owing to the weak van der Waals interlayer bonding. Figure 3f presents the thermal conductivity anisotropic ratio *k*_zigzag_/*k*_armchair_, which drops from ∼2 to ∼1.5, as BP thickness decreases to <10 nm. It is worth noting that the error bars in Fig. 3f did not account for the uncertainty of *k*_SiN_, as any variation in SiN substrate thermal conductivity will affect both *k*_armchair_ and *k*_zigzag_, which cannot be treated by standard error propagation method, as it requires uncertainties to be mutually independent.

**Figure 4:**
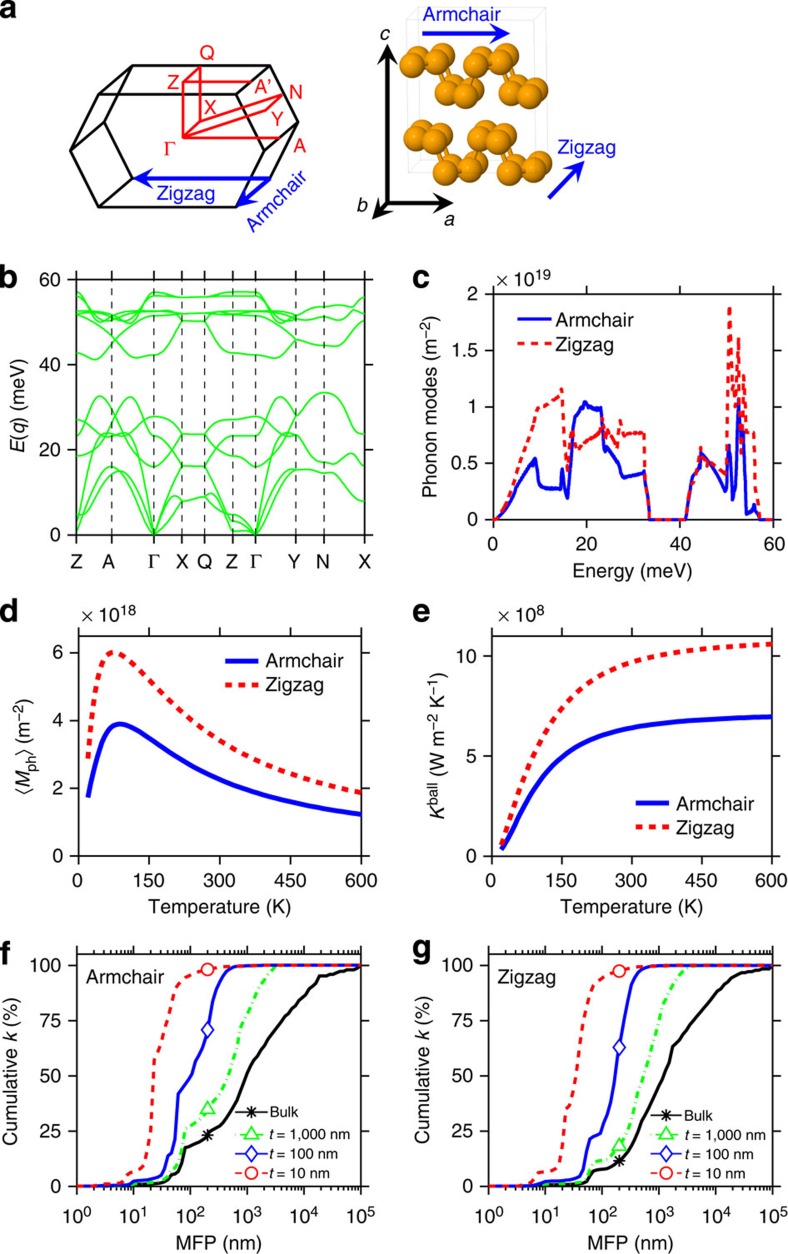
First-principles-based modelling results of few-layer BP. (**a**) High symmetry points in the Brillouin zone (left) and crystal structure (right) of black phosphorus. (**b**) Phonon dispersion (energy *E* versus momentum *q*) along high symmetry points. (**c**) Number of conducting phonon modes per cross-sectional area versus energy. (**d**) Average number of thermally active phonon modes per cross-sectional area as a function of temperature. (**e**) Ballistic thermal conductance as a function of temperature. (**f**,**g**) Normalized cumulative thermal conductivity at 300 K versus phonon MFP for backscattering (using phenomenological scattering models for Umklapp and surface scattering with specularity parameter *p*=0) for transport along armchair and zigzag directions.

Theoretical modelling of few-layer BP, based on *ab initio* phonon dispersion calculations and phenomenological scattering models, was conducted to understand the experimental results. The phonon dispersion of bulk BP (valid for our thin films, see Supplementary Fig. 4 and Supplementary Note 6) along the high-symmetry reciprocal lattice points (Fig. 4a) was calculated with the optimized lattice constants given as *a*=4.57 Å, *b*=3.30 Å and *c*=11.33 Å (ref. 41), and the results are shown in Fig. 4b. While our structural optimization yields lattice constants that are slightly different than those reported in ref. 41 (Supplementary Note 7), the electron and phonon dispersions do not show significant differences (Supplementary Figs 5,6). There are three acoustic branches and nine optical branches extending up to roughly 55 meV, with an energy gap between 35 and 40 meV. Significant differences of acoustic phonon bandwidths and group velocities are observed between Γ–X (armchair) and Γ–A (zigzag) (and also Γ–Z cross-plane) directions, which are responsible for the measured thermal conductivity anisotropy. We then computed the transport properties solving the phonon Boltzmann equation using the Landauer approach^39^,^42^, which expresses the thermal conductivity as


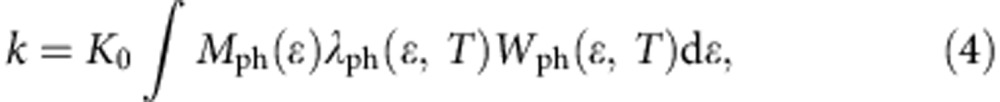


where *K*_0_=*π*^2^*k*_B_^2^*T*/3*h* is the quantum of thermal conductance, *M*_ph_ is the number of conducting modes per cross-sectional area (units: m^−2^), *λ*_ph_ is the phonon MFP for backscattering, which includes Umklapp phonon–phonon scattering and surface scattering, and *W*_ph_(*ɛ*,*T*)=(3*ɛ*/*π*^2^*k*_B_^2^*T*)[∂*n*_BE_(*ɛ*,*T*)/∂*T*] is a normalized ‘window function' with *n*_BE_ being the Bose–Einstein distribution and *ɛ* the phonon energy. It can be seen that the above formula of thermal conductivity consists of two quantities: the number of phonon modes per cross-sectional area *M*_ph_, which depends only on the phonon dispersion, and the phonon MFP *λ*_ph_, which depends on both the phonon dispersion and the scattering mechanisms. *M*_ph_ can be readily and efficiently calculated using the ‘band-counting' algorithm as implemented in the simulation tool LanTraP^43^. The calculated phonon modes *M*_ph_ along the armchair and zigzag directions are presented in Fig. 4c, where *M*_ph_ along the zigzag direction shows a significantly larger number of modes, in particular for the acoustic phonons in the energy range 5–15 meV, which typically carry most of the heat. This anisotropy in number of modes is related to the larger phonon group velocity along the zigzag direction compared with the armchair direction, as observed from the slopes of the phonon dispersion. At a given temperature, the average number of thermally active phonon modes is given by^42^


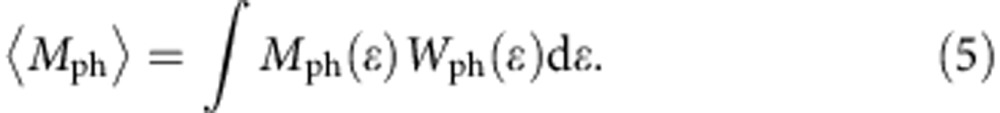


In the absence of scattering, the ballistic thermal conductance *K*^ball^, which is independent of sample length, is simply 
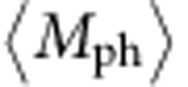
 times the quantum of thermal conductance *K*_0_. The calculated temperature-dependent 
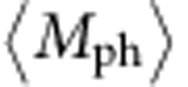
 and *K*^ball^ are shown in Fig. 4d,e, and they display a strong anisotropy between zigzag and armchair directions (it is noteworthy that the calculated phonon dispersion is extrapolated to other temperatures, as we assume it does not significantly change in the plotted temperature range). Above 150 K, their respective zigzag-to-armchair ratio is almost constant and equal to ∼1.5. Given that the thermal conductivity *k* can be written as *K*^ball^ times the average phonon MFP 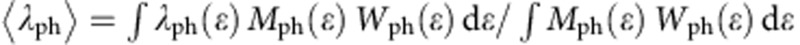 (here we define an average phonon MFP to help explain our results; it is important to note that the full MFP distribution is used in our theoretical calculations, which can span orders of magnitude), the thermal conductivity ratio *k*_zigzag_/*k*_armchair_ can be expressed as 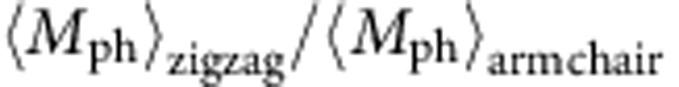 times 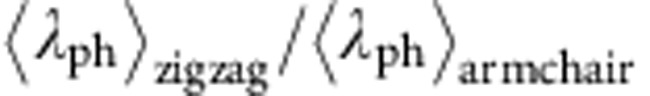. This suggests that the ∼1.5–2.0 anisotropic ratio of measured thermal conductivity is in large part due to 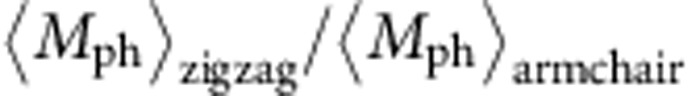, a quantity that depends only on the phonon dispersion and shows a zigzag-to-armchair ratio of ∼1.5 at room temperature. As the BP film thickness decreases, the minor contribution of 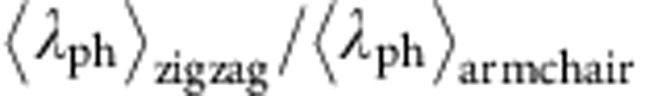 (that is, the anisotropy in phonon MFP) further decreases, owing to the enhanced surface scattering. Thus, the thermal conductivity anisotropic ratio *k*_zigzag_/*k*_armchair_ decreases and approaches ∼1.5, as depicted in Fig. 3f.

To calculate the in-plane thermal conductivity of few-layer BP films, the phonon MFP distribution *λ*_ph_(*ɛ*,*T*) in equation (4) must be specified. We included intrinsic Umklapp phonon–phonon scattering and surface scattering of phonons at film boundaries to determine the phonon MFP distribution. Each scattering mechanism, Umklapp and surface scattering, has one free parameter that can be adjusted to fit the measured thermal conductivity (see Supplementary Note 7 for further details). Using the Fuchs–Sondheimer^39^,^42^ approach to include the effect of surface scattering in few-layer BP, the strength of the surface scattering is controlled through a specularity parameter *p*, which dictates the degree to which scattering at the surface is specular (denotes an atomically smooth surface where momentum along the transport direction is conserved) or diffusive (denotes a rough surface where momentum along the transport direction is randomized). We note that this surface scattering model was originally developed assuming isotropic bulk scattering and it was adopted in this work to investigate anisotropic BP as the best available candidate. Our theoretical model provides a good match to the measured thermal conductivity with a specularity parameter *p* in the range 0–0.4 (Supplementary Fig. 7), shown as dashed curves in Fig. 3e (with *p*=0). This is consistent with the fact that few-layer BP is susceptible to chemical reactions with oxygen and moisture^14^, as well as possible minor poly(methyl methacrylate) (PMMA) residue (even undetectable with Raman spectroscopy and AFM), which may have enhanced surface scattering. Interestingly, the intrinsic Umklapp scattering rate distribution used to calculate *k* in Fig. 3e is the same along both armchair and zigzag directions. With an isotropic scattering rate, the intrinsic phonon MFP is longer along the zigzag direction compared with the armchair direction (that is, anisotropic), due to the difference in phonon group velocities. Our theoretical analysis suggests that the measured thermal conductivity anisotropy is primarily a consequence of the particular phonon dispersion of BP, as opposed to strong anisotropic scattering of phonons. Even with an isotropic MFP, the thermal conductivity remains anisotropic (Supplementary Fig. 8). Previous first-principles ballistic calculations of monolayer BP, which only consider phonon dispersion, also display significant anisotropy in the thermal transport properties^27^.

To further illustrate how phonons of different MFP contribute to the thermal conductivity and the impact of surface scattering, in Fig. 4f,g we plot the normalized cumulative *k* versus MFP at different BP film thicknesses. They were obtained using the first-principles-calculated phonon dispersion and the phenomenological scattering models, which were calibrated to the measured BP film thermal conductivities, as described above. It can be clearly seen how surface scattering strongly alters the contribution of long-MFP phonons. For example, in a 10-nm-thick film, nearly 90% of the heat is carried by phonons with MFP <100 nm, as opposed to roughly 20% and 10% in bulk along armchair and zigzag directions, respectively. It is worth noting that the plots of cumulative *k* versus MFP for thicknesses >30 nm are an extrapolation. For bulk BP a significant contribution to *k* comes from phonons with MFP around 1 μm, whereas for 10-nm-thick BP film the largest contribution comes from phonons with MFP near 30 nm. Therefore, for thermoelectric applications where higher electro-thermal conversion efficiency results from lower *k*, further reductions in few-layer BP thermal conductivity could be achieved by selectively scattering phonons that contribute the most^44^. However, in cases where a large thermal conductivity is beneficial (for example, to mitigate hotspots in electronic devices), thicker BP films are desirable because of less surface scattering. Alternatively, finding solutions to enhance surface smoothness and reduce diffusive surface scattering, such as encapsulating BP by an inert hBN layer^45^, could be equally effective.

In summary, we experimentally measured the anisotropic in-plane thermal conductivity of suspended few-layer BP films using micro-Raman spectroscopy and performed first-principles-based theoretical studies to gain insight into the anisotropic thermal transport. The armchair and zigzag thermal conductivities, *k*_armchair_ and *k*_zigzag_, are ∼20 and ∼40 W m^−1^ K^−1^ for BP films over 15-nm thickness, respectively. Strong surface scattering of phonons reduces *k*_armchair_ and *k*_zigzag_ by half, down to ∼10 and ∼20 W m^−1^ K^−1^, respectively, for the thinnest 9.5-nm-thick BP film. The thermal conductivity anisotropic ratio *k*_zigzag_/*k*_armchair_ is found to be ∼2 for thick BP films and drops to ∼1.5 for the thinnest one. Theoretical modelling of thermal transport in few-layer BP reveals that the observed anisotropic thermal conductivity stems from the material's particular phonon dispersion, as opposed to anisotropic scattering. Diffusive surface scattering is found to be prevalent in few-layer BP, which strongly reduces the contribution of long MFP phonons. Our results may provide useful guidance for the design of BP-based thermoelectric, electronic and optoelectronic devices from the perspective of anisotropic thermal transport.

During this study, we became aware of recently published angle-dependent Raman spectroscopy works on BP, which exploited the same polarized Raman technique^46^,^47^.

## Methods

### Flake preparation and transfer

On the Si wafer, ∼1-μm-thick poly(vinyl alcohol) (PVA) was spin-coated and baked at 70 °C for 5 min and ∼200-nm-thick PMMA (950 A4) was spin-coated onto PVA and baked using the same recipe. Silicon dicing tape was used to peel flakes off the bulk BP crystal, then the flakes were released on to the PMMA/PVA stack. The polymer stack was then cleaved off, flipped over and mounted on a glass frame for subsequent visual examination under microscope. Once a candidate flake was found, polarized Raman measurements were conducted to identify the lattice orientation. Then the flake along with the PMMA/PVA was aligned in the desired direction and attached to the slits fabricated by focused-ion-beam (FEI Nova) on the 200-nm-thick free-standing SiN substrate film. The whole sample was then dipped into acetone to dissolve the PMMA layer and to remove the PVA layer, then finally dried with nitrogen. Supplementary Fig. 2 illustrates the process. We used a large amount of acetone (> 70 ml) and very long soaking time (> 12 h) to minimize the PMMA residue on the BP films, and the surface cleanliness was confirmed by AFM scans and Raman spectra. The BP flakes were not baked or annealed throughout the preparation and transfer process, to avoid excessive oxidation and to retain the crystallinity. During the flake preparation and transfer process, the BP flake was exposed to the air for about 1 h, while all the subsequent Raman measurements were performed in dry nitrogen atmosphere.

### Micro-Raman and SEM/AFM measurements

A HORIBA LabRAM HR800 Raman spectrometer equipped with a 632.8-nm wavelength He-Ne laser and an Olympus × 100 objective lens was used for all the Raman measurements. The nominal spectral resolution is 0.27 cm^−1^ per pixel and the Lorentzian peak fitting yields a peak position shift uncertainty <0.02 cm^−1^, corresponding to a temperature rise measurement uncertainty of <1 K for BP. A linear polarizer (Thorlabs, LPNIRE050-B) was used for polarized Raman measurements. The SEM images were taken by an FEI Nova system and the AFM measurements were carried out on an AIST-NT CombiScope system.

### Laser focal line characterization

Along the length direction, the intensity profile *I*_*l*_ was extracted directly from the raw red, green, and blue (RGB) data of a CCD (charge-coupled device) camera and fitted with Gaussian function to obtain *l*_0_


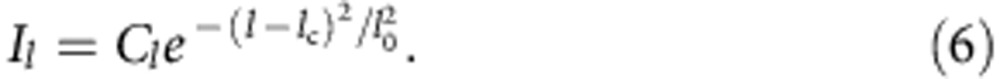


We performed knife-edge measurements to characterize the width of the focal line. A Si wafer with sharp edge was gradually brought into the focal line along the width direction by a piezoelectric stage. As the stage moved the Raman intensity of Si increased, as more Si was exposed to the laser. Similar to the length direction, the intensity profile along the width direction *I*_*w*_ can also be described by a Gaussian function


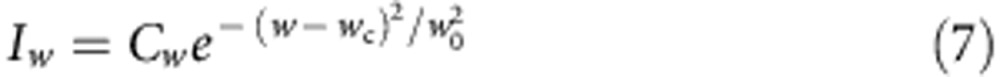


and the Raman intensity of Si as a function of stage position is the integration of *I*_*w*_ along the width direction


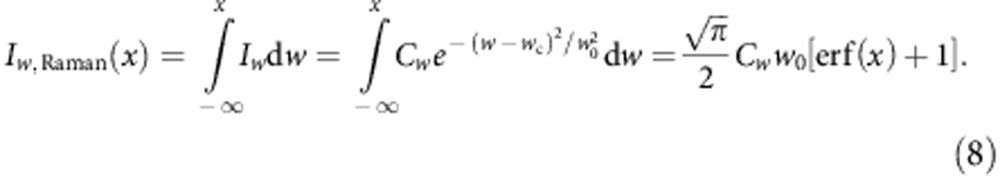


By fitting the measured intensity into the above equation, we were able to obtain *w*_0_.

## Additional information

**How to cite this article:** Luo, Z. *et al.* Anisotropic in-plane thermal conductivity observed in few-layer black phosphorus. *Nat. Commun.* 6:8572 doi: 10.1038/ncomms9572 (2015).

## Supplementary Material

Supplementary Information Supplementary Figures 1-8, Supplementary Notes 1-7 and Supplementary References
