# Supplementary material for: Anisotropic in-plane thermal conductivity observed in few-layer black phosphorus
Source: Nat Commun. 2015 Oct 16;6:8572. doi: 10.1038/ncomms9572 (PMC4634212; doi:10.1038/ncomms9572)
Supplement: Supplementary Information — Supplementary Figures 1-8, Supplementary Notes 1-7 and Supplementary References [file ncomms9572-s1.pdf]

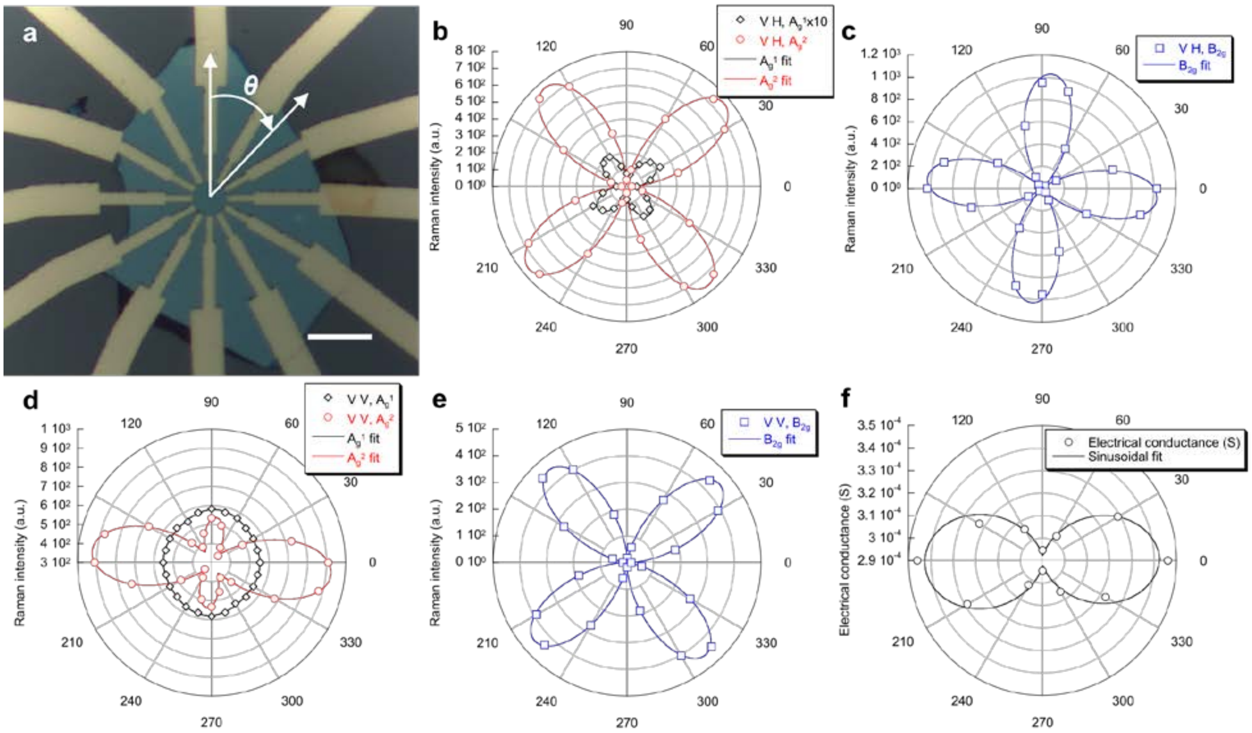

Supplementary Figure 1 | Polarized-Raman and electrical conductance measurements on a 32-nm-thick black phosphorus (BP) flake. (a) Optical image showing the flake and the electrodes. Scale bar is 10 μm. Angle-resolved polarized Raman intensity of (b) A_g modes (c) B_{2g} mode in VH configuration, and (d) A_g modes (e) B_{2g} mode in VV configuration. In (b) to (e), solid lines are all curve fits based on Supplementary Equation 3. (f) Angle-resolved electrical conductance measured by six pairs of electrodes using 50 mV voltage. Solid line is the sinusoidal curve fit.

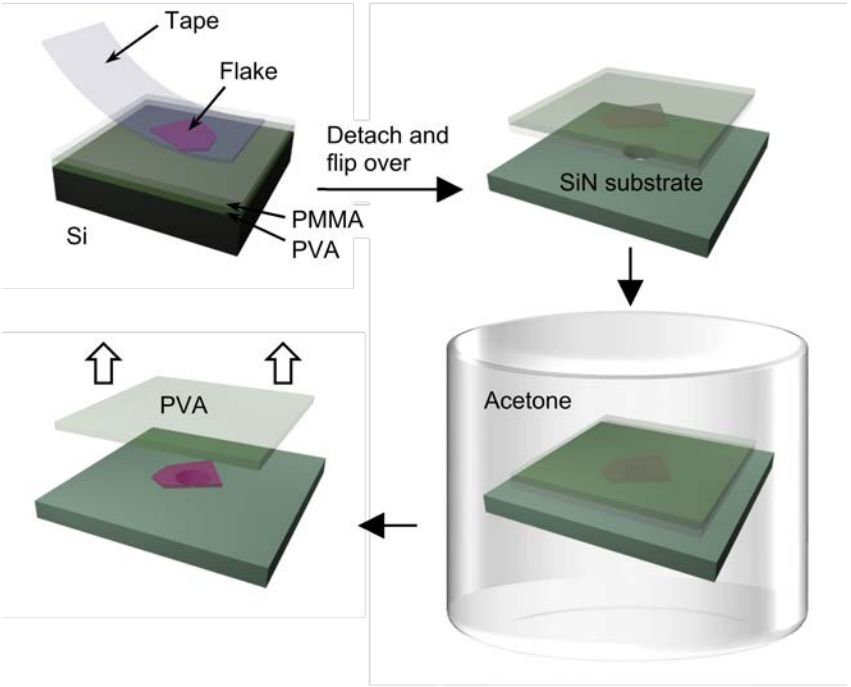

Supplementary Figure 2 | Sketch of the flake preparation and transfer process.

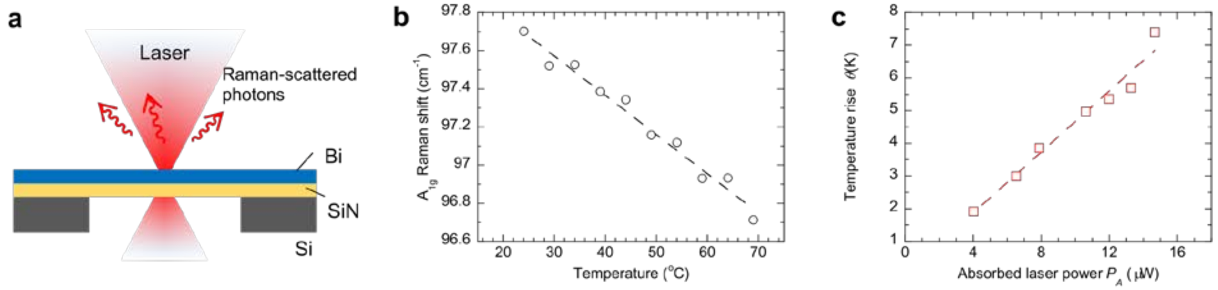

Supplementary Figure 3 | Micro-Raman measurement of the Bi-SiN sample. (a) Sketch of the sample structure and experimental setup. (b) A_{1g} Raman thermometer calibration results. Dashed line is the linear fit showing temperature coefficient $\chi_{A_{1g}} = -0.0206$ cm^{-1}. (c) Laser-power-dependent temperature rise, and the linear fit with slope $d\theta/dP_A = 0.4671$ K μW^{-1}. The Raman-measured temperature uncertainty is ~1 K.

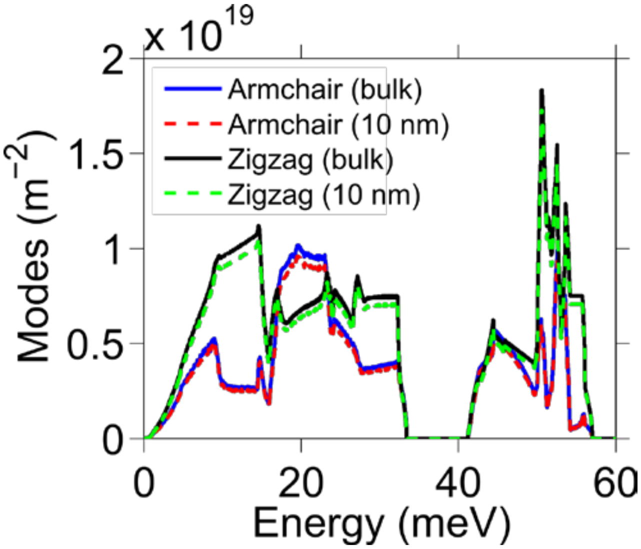

Supplementary Figure 4 | Impact of confinement on number of phonon modes in BP. Number of modes per cross-sectional-area M_{ph} versus phonon energy for bulk BP and a 10-nm-thick BP film. The number of modes in the film was calculated assuming that phonons with half-wavelength (in the cross-plane direction) greater than the film thickness do not contribute to the number of modes.

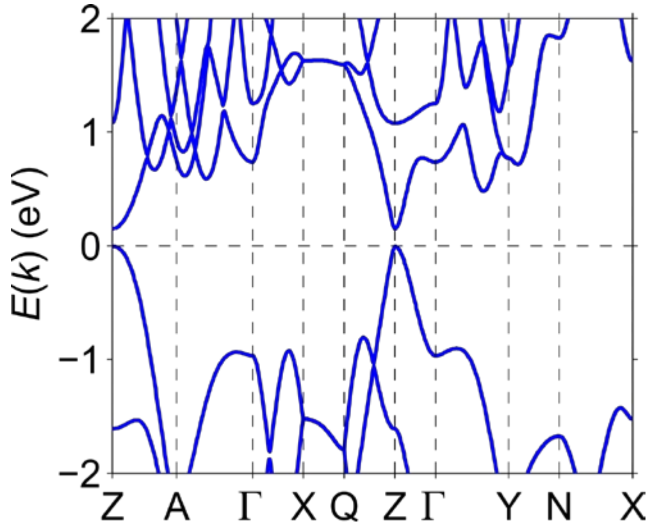

Supplementary Figure 5 | Electron dispersion of bulk BP. Electron band structure of bulk BP along high symmetry points.

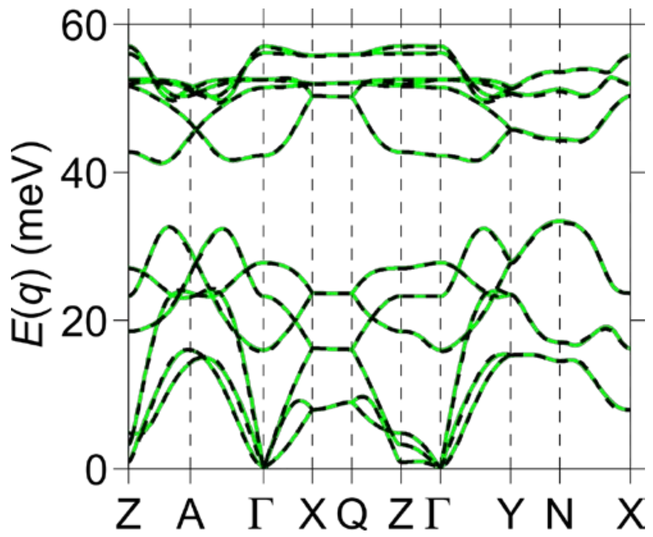

Supplementary Figure 6 | Phonon dispersion of bulk BP. Comparison of phonon dispersion along high-symmetry points using the lattice constants reported in *Nat. Commun.* **5**, 4475 (2014) *a* = 4.57 Å, *b* = 3.30 Å, *c* = 11.33 Å (solid black lines) and our total-energy minimized lattice constants *a* = 4.56 Å, *b* = 3.31 Å, *c* = 11.32 Å (dashed green lines).

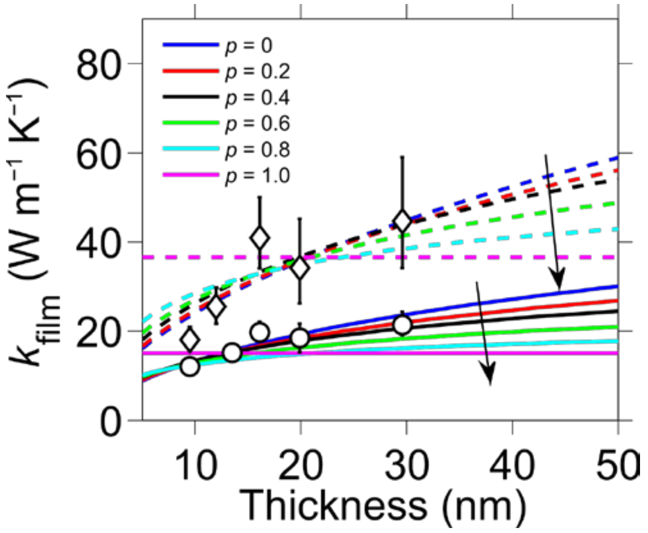

Supplementary Figure 7 | Effect of specularity parameter *p*. Thermal conductivity versus BP film thickness for zigzag (diamonds, dashed lines) and armchair (circles, solid lines) directions, as a function of specularity parameter *p*. For a given *p* value, the *B* parameter controlling the strength of the Umklapp scattering is adjusted to best match the measured values (symbols). For *p* ranging from 0 to 1 with 0.2 increments, the optimal *B* was found to be 2×10^{-19} s K^{-1}, 3×10^{-19} s K^{-1}, 4.2×10^{-19} s K^{-1}, 6.3×10^{-19} s K^{-1}, 9.5×10^{-19} s K^{-1}, 1.6×10^{-18} s K^{-1}, respectively. The arrows indicate the trend for increasing *p*.

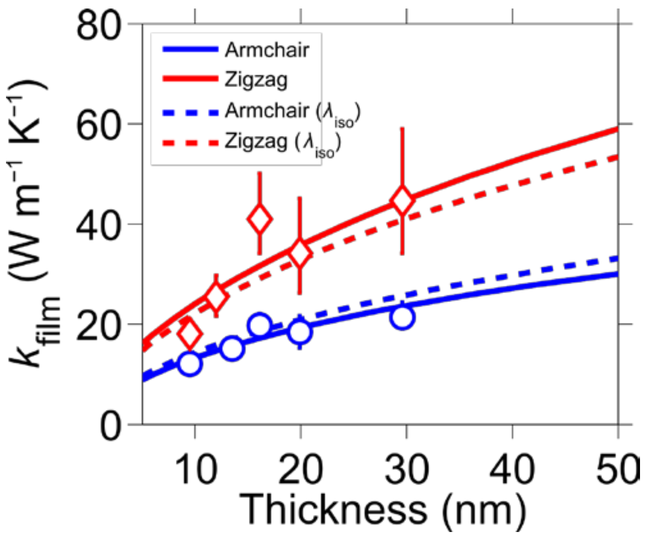

Supplementary Figure 8 | Anisotropic versus isotropic phonon mean-free-path (MFP). Thermal conductivity versus BP film thickness along armchair and zigzag directions. Markers are experimental data. Solid lines are calculated using an anisotropic MFP distribution (i.e. isotropic scattering time distribution), as described by our methods. Dashed lines are calculated using an isotropic MFP distribution obtained by averaging the anisotropic MFP over armchair and zigzag directions at each energy. In the case of an isotropic MFP, significant anisotropy in thermal conductivity is observed arising from the anisotropy in the phonon modes.

Supplementary Note 1 | Polarized-Raman measurements

The Raman scattering intensity I is proportional to[1]

$$I \propto \left|\mathbf{e}_\mathrm{i} \times \mathbf{R} \times \mathbf{e}_\mathrm{s}\right|^2, \tag{1}$$

where $\mathbf{e}_\mathrm{i}$, $\mathbf{e}_\mathrm{s}$ represent incident and scattered light polarizations, respectively, and $\mathbf{R}$ is the Raman tensor of a specific Raman mode. In BP lattice *abc* coordinates where *a* is the zigzag direction, *b* is the cross-plane direction and *c* is the armchair direction, the incident laser polarization is expressed as $\mathbf{e}_\mathrm{i}$ = (cosθ, 0, sinθ) where θ is the angle between the *a* axis and the laser polarization vector (Supplementary Fig. 1a), and the detection polarization $\mathbf{e}_\mathrm{s}$ is set as $\mathbf{e}_\mathrm{s} \perp \mathbf{e}_\mathrm{i}$ (VH configuration) or $\mathbf{e}_\mathrm{s} \parallel \mathbf{e}_\mathrm{i}$ (VV configuration). For BP, three Raman-active modes A_g^1 B_{2g} and A_g^2 can be observed, and their Raman tensors are[2]

$$\mathbf{R}_{\mathrm{A_g}} = \begin{pmatrix} A & & \\ & B & \\ & & C \end{pmatrix}, \quad \mathbf{R}_{\mathrm{B_{2g}}} = \begin{pmatrix} & & E \\ & & \\ E & & \end{pmatrix}, \tag{2}$$

then the Raman intensity can be written as

$$\text{VH:}\begin{cases} I_{\mathrm{A_g}} \propto \dfrac{(C-A)^2}{4}\sin^2 2\theta \\ I_{\mathrm{B_{2g}}} \propto E^2\cos^2 2\theta \end{cases} \quad \text{VV:}\begin{cases} I_{\mathrm{A_g}} \propto \left(A\cos^2\theta + C\sin^2\theta\right)^2 \\ I_{\mathrm{B_{2g}}} \propto E^2\sin^2 2\theta \end{cases}. \tag{3}$$

When incident laser polarization is along the zigzag direction (θ = 0°),

$$\text{VH:}\begin{cases} I_{\mathrm{A_g}} = 0 \\ I_{\mathrm{B_{2g}}} \propto E^2 \end{cases} \quad \text{VV:}\begin{cases} I_{\mathrm{A_g}} \propto C^2 \\ I_{\mathrm{B_{2g}}} = 0 \end{cases}, \tag{4}$$

and when incident laser polarization is along the armchair direction (θ = 90°),

$$\text{VH:}\begin{cases} I_{\mathrm{A_g}} = 0 \\ I_{\mathrm{B_{2g}}} \propto E^2 \end{cases} \quad \text{VV:}\begin{cases} I_{\mathrm{A_g}} \propto A^2 \\ I_{\mathrm{B_{2g}}} = 0 \end{cases}. \tag{5}$$

It can be clearly seen that $I_{\mathrm{A_g}}$ in VV configuration can be used to distinguish zigzag and armchair axes. Supplementary Figure 1 presents collected polarized Raman intensity from a 32-nm-thick BP flake and sinusoidal fitting using Supplementary Equation 3, showing clear angle-dependent signature. Note that for A_g^1 mode Raman tensor, A = C, so that only A_g^2 mode can be used to determine the lattice orientation (armchair where intensity gets maximized) as shown in Supplementary Fig. 1d. To further confirm the lattice orientation, six pairs of 0.3 nm Ti/40 nm

Pd/40 nm Au electrodes were patterned with 30° interval on the flake using electron beam lithography to measure angle-dependent electrical conductivity, and the results are shown in Supplementary Fig. 1f. It has been demonstrated that electrical conductance is higher along the armchair direction[3], so that our electrical measurement validates the lattice orientation derived from polarized-Raman measurements.

Supplementary Note 2 | Excessive strain and stress effects on the Raman-based thermal measurements of BP films

Raman shift is known to be sensitive to strain and stress. In our micro-Raman experiments, the heating-induced thermal strain contributes to a part of the temperature coefficient and is calibrated during the calibration processes. The only undesirable strain or stress comes from the thermal expansion mismatch between the BP film and the SiN membrane during heating. Since the thermal expansion of BP α_{BP} ~50 × 10^{-6} K^{-1} (ref. 4) is larger than that of SiN α_{SiN} ~2 × 10^{-6} K^{-1} (ref. 5), a compressive stress will possibly be applied to the BP film due to the constraint of the SiN membrane. However, due to the large aspect ratio of the BP film (< 30 nm thickness vs. 3 μm width), the compressive stress is expected to be relaxed by natural buckling and thus does not affect our Raman measurements.

To further evaluate this stress effect, we conducted separate calibrations on both suspended regions (two perpendicular slits) of the ~20-nm-thick BP film. The compressive stress on the two regions, if any, would be perpendicular to the slits and therefore orthogonal to each other. Since the thermal expansion and the Raman-strain response of BP are anisotropic[6], the orthogonal compressive stress would lead to different temperature coefficient if such stress plays a significant role. The obtained temperature coefficients on the two regions are −0.0260 cm^{-1} K^{-1} and −0.0268 cm^{-1} K^{-1}, respectively, with only a minute relative difference of ~3%. This indicates that the suspended regions of BP films remain free from excessive strain and stress that originate from the constraint of SiN membrane during heating, thus we believe that the compressive stress due to thermal expansion mismatch has a negligible effect on our Raman measurements.

It is worth noting that there are other Raman indicators such as peak linewidth and anti-Stokes to Stokes intensity ratio which can serve as temperature probes and are insensitive to strain and stress; however, they are not as accurate as the Stokes peak shift[7,8]. Trade-offs between stress-sensitivity and temperature-accuracy should be carefully considered, based on the instrument capability and samples to be investigated. If Raman peak shift thermometer is to be implemented, the strain and stress effects should be carefully evaluated.

Supplementary Note 3 | Determination of k_{armchair} and k_{zigzag} and their uncertainties

Although the heat transfer in suspended BP films is close to 1D, k_{armchair} and k_{zigzag} are still weakly coupled. Therefore we extract them using the iterative calculations using our numerical heat transfer model. For a given flake, it is suspended on two perpendicular slits for measuring k_{zigzag} and k_{armchair} separately, which are referred to as ZZ slit and AC slit here for convenience. First we assumed a k_{zigzag} and applied it to the modeling of the AC slit to extract a k_{armchair}; then the extracted k_{armchair} was in turn applied to the modeling of the ZZ slit and a new k_{zigzag} was obtained. Then the previously assumed k_{zigzag} was replaced by this new value, and the above process was iteratively repeated until both k_{armchair} and k_{zigzag} converge.

The uncertainties of k_{armchair} and k_{zigzag} are determined using the standard error propagation method, i.e. $\sigma_k = [\Sigma(A_i\sigma_i)^2]^{1/2}$ where σ_i is the uncertainty of a single parameter and A_i represents the linearized effect this parameter has on the final result. We have included all uncertainties of concern such as that of data slope $\mathrm{d}\theta/\mathrm{d}P$, temperature coefficient χ, thickness t, absorptivity A, and SiN thermal conductivity k_{SiN}. Since the relative uncertainties of $\mathrm{d}\theta/\mathrm{d}P$, χ, t and A are the same or approximately the same for the two directions for a given flake, the relative uncertainty of k, which is σ_k/k, remains approximately the same as well, according to error propagation theory. This yields $\sigma_{k,\mathrm{zigzag}}/\sigma_{k,\mathrm{armchair}} \sim k_{\mathrm{zigzag}}/k_{\mathrm{armchair}} \sim 1.5–2$, which explains the larger colored error bars of k_{zigzag} in Fig. 3e. For gray error bars, we took into consideration the uncertainty of k_{SiN}. It has larger impact on the uncertainty of k_{zigzag} than k_{armchair} as seen in Fig. 3e. The reason is that, in zigzag measurements, the temperature distribution across the slit is more flat due to the higher thermal conductivity in the zigzag direction, therefore the temperature is higher at the edge of the slit so that the conductivity of the heat sink (SiN) plays a bigger role than the

armchair case. This means that $\sigma_{k,\mathrm{zigzag}}$ is more sensitive to $\sigma_{k,\mathrm{SiN}}$ than $\sigma_{k,\mathrm{armchair}}$, which explains the even longer gray error bars of k_{zigzag}.

Supplementary Note 4 | Evaluation of the convective heat transfer loss, the heat sink efficiency, and measurement of SiN thermal conductivity k_{SiN}

To minimize the convective heat transfer loss due to the nitrogen flow, the flow rate is controlled at a minimum level of ~7 cm^3 s^{-1}. Considering the cross-sectional area of a few cm^2 of our measurement chamber, the nitrogen flow speed at the sample surface would be on the order of 1 cm s^{-1}, so that the effect of the convection was close to that of free convection. To further quantify the convection effect, we performed numerical analysis on the thinnest 9.5 nm flake which would likely show largest convective heat transfer loss due to its largest surface-to-volume ratio. Typical free convective heat transfer coefficient ranges from 2–25 W m^{-2} K^{-1} (ref. 9). We calculated h = 0 W m^{-2} K^{-1} and h = W m^{-2} K^{-1} cases and found the armchair (zigzag) thermal conductivity to be 12.14 (18.22) W m^{-1} K^{-1} and 11.99 (17.94) W m^{-1} K^{-1}, respectively. The relative difference due to convective heat transfer loss is only ~1.5%. h = 20 W m^{-2} K^{-1} was implemented in our modeling as the best estimate, which would lead to an even smaller difference in the thermal conductivity values.

We also analyzed the heat conduction through a 200-µm-thick layer of N_2 between the BP film and the sample holder and the heat conduction in N_2 from the top of the BP film to the objective lens through a distance of 210-µm, the working distance of the objective lens. Numerical calculations on the 9.5-nm-thick BP film found that the extracted armchair and zigzag thermal conductivity to be 11.77 and 17.42 W m^{-1} K^{-1}, respectively. Comparing with the case considering convection only with h = 20 W m^{-2} K^{-1}, in which the armchair and zigzag thermal conductivity are 12.06 and 18.08 W m^{-1} K^{-1} respectively, the relative difference is less than 3.8%. Note that for thicker BP films this discrepancy will be smaller, since the heat conduction within BP films will become more prominent.

To assess the effects of cross-plane heat conduction in the supported region of the BP films, we performed sensitivity studies on three key parameters: the cross-plane thermal conductivity of

BP $k_{z,BP}$, the contact thermal resistance at BP/SiN interface R_c and the thermal conductivity of SiN membrane k_{SiN}. It turns out that $k_{z,BP}$ and R_c do not affect much the fitted BP thermal conductivity. Varying $k_{z,BP}$ from 0.5 to 5 W m^{-1} K^{-1}, which is considered typical for vdW bonds[10], only resulted in ~1% difference of the extracted BP thermal conductivity; varying R_c from 1×10^{-9} to 1×10^{-7} m^2 K W^{-1}, typical for vdW interfaces[11], gave ~4.5% difference. In contrast, k_{SiN} has a larger impact, as a variation from 8 to 10 W m^{-1} K^{-1} (intermediate value in literature reported range[12–14]) leads to as much as 50% change of the fitted BP thermal conductivity. Hence, we conducted separate micro-Raman measurements to measure k_{SiN} using thermally evaporated bismuth (Bi) film as the Raman transducer, which has been validated by the successful measurements of amorphous Al_2O_3 thin films[15].

Bi (99.999% purity, Sigma Aldrich) was thermally evaporated onto fresh 200-nm-thick SiN membrane (Product# 21520, Ted Pella) at the vacuum level of ~1×10^{-6} Torr. A clean glass slide was coated simultaneously, and the Bi film on glass was scratched intentionally so that the Bi film thickness could be determined using AFM. The thickness was measured to be 34.7 ± 1.3 nm. Then the same micro-Raman technique, except that the focal spot was circular instead of line-shaped, was utilized on the Bi-SiN film stack as sketched in Supplementary Fig. 3a. The Bi-SiN sample was calibrated (Supplementary Fig. 3b) and measured (Supplementary Fig. 3c) using A_{1g} mode (~97 cm^{-1}) as the Raman thermometer. The slope $d\theta/dP_A$ was fitted into a 2D radial heat transfer model where the thermal conductivity of Bi k_{Bi} was taken from our previous work (8.4 ± 0.9 W m^{-1} K^{-1})[15]. Note that the Bi films were prepared under the same condition, so that the use of previous obtained k_{Bi} was considered valid. By fitting the model, k_{SiN} is extracted to be 9.4 + 1.3/−1.1 W m^{-1} K^{-1} which is used for extracting the thermal conductivity of BP, along with $k_{z,BP}$ taken as 1 W m^{-1} K^{-1} and R_c taken as 2×10^8 m^2 K W^{-1}. The absorptivity of the Bi-SiN sample was measured in the same way as described in the "Results" Section of the main text. The uncertainty of measured k_{SiN} was determined using the error propagation method (see Supplementary Note 3) and propagated into the uncertainty of measured BP thermal conductivity. It is noted that k_{SiN} reported in the literature ranges from 1–13 W m^{-1} K^{-1}, which we found might be related to the composition of the SiN films[12,14,16–18]. With Si composition increasing, k_{SiN} increases and reaches > 8 W m^{-1} K^{-1} for Si:N = 1:0.99 (ref. 14). The Si:N atomic ratio of the SiN films used in this study is a rather high 1:0.92, thus justifying the k_{SiN} measured to be 9.4 W m^{-1} K^{-1} which falls into the higher side of the reported range.

We also attempted to measure k_{SiN} using the time-domain thermoreflectance (TDTR) method. A 150-nm-thick Au layer was coated on the SiN sample, so that the film stack structure from top to bottom is: 150 nm Au, 200 nm SiN, 240 nm SiO_2, bulk (200 μm) Si. A standard TDTR setup equipped with a 532-nm Nd:YAG pulsed laser (~5 ns pulsewidth, 5 kHz repetition rate) as the pump laser and a 633-nm He-Ne continuous wave laser as the probe laser was used to obtain the data. The free parameters were k_{SiN}, and the contact resistances of the Au-SiN (R_1), SiN-SiO_2 (R_2), and SiO_2-Si (R_3) interfaces. All the other parameters were taken from widely used bulk values. However, because the contact resistance values are unknown, k_{SiN} was not conclusively determined. With all contact resistances varied by only ~20%, we were able to fit the experimental data with both k_{SiN} = 5 W m^{-1} K^{-1} and 10 W m^{-1} K^{-1}.

Supplementary Note 5 | Estimating the electronic contribution to the thermal conductivity

To estimate the electronic thermal conductivity of BP, we again used the Landauer approach[31]. By following essentially the same procedure outlined above for phonons, we extracted the electron dispersion from DFT (the scissor method was employed to adjust the band gap to the experimentally accepted value 0.33 eV), computed the number of modes per cross-sectional-area for electrons, in which a constant electron MFP was used. The equations relating number of modes and MFP to electrical conductivity, Seebeck coefficient, and electronic thermal conductivity are provided in ref. 22. In ref. 3, the authors measured the hole mobility of a 8-nm-thick BP film at room temperature to be ~420 cm^2 V^{-1} s^{-1} and ~270 cm^2 V^{-1} s^{-1} along the armchair and zigzag directions, respectively. Using the measured hole concentration of ~1.7 × 10^{19} cm^{-3} combined with our calculation of carrier concentration from the density-of-states, we determined that the Fermi level is located at 0.06 eV into the valence band. By fitting the MFP for electrons along armchair and zigzag to reproduce the mobility values, we found $\lambda_{armchair}$ = 13 nm and λ_{zigzag} = 38 nm. From this we can calculate the electronic thermal conductivity: ~0.65 W m^{-1} K^{-1} (armchair) and ~0.45 W m^{-1} K^{-1} (zigzag). The lattice contribution to the thermal conductivity is significantly larger than the electronic contribution, and the latter can be neglected in the analysis of the measured thermal conductivity.

Supplementary Note 6 | Impact of confinement on the phonon dispersion

In the theoretical calculations we assumed that the phonon dispersion of bulk BP represents well the phonon states in the experimental few-layer BP films, since calculating the phonon dispersion for each film thickness would be prohibitively demanding. In order to validate this assumption, we computed the number of modes per cross-sectional-area M_{ph} assuming that all phonons with half-wavelength greater than the film thickness do not contribute to the number of modes to estimate the impact of confinement. With this calculation, we can estimate the proportion of phonons which have wavelengths similar to the thickness therefore would likely be affected by the confinement. Supplementary Figure 4 compares M_{ph} versus energy for bulk BP and a 10-nm-thick film. Only minor differences are observed, justifying our model using the phonon dispersion of bulk BP.

Supplementary Note 7 | Calculation of lattice thermal conductivity

We computed the thermal transport properties of few-layer BP using the Landauer approach, which has been successfully used to describe phonon transport in thin films[19,20]. Within this approach the lattice thermal conductivity k is expressed in equation (4) in the main article, where we can see that the two main quantities needed to obtain the thermal conductivity are the number of modes per cross-sectional-area M_{ph} and the phonon MFP λ_{ph} (referred to as λ_{film} below in order to distinguish from the phonon MFP of bulk BP).

M_{ph} is calculated using equation (A4) in ref. 21 or equivalently equation (16) in ref. 22, which depends only on phonon dispersion. The phonon dispersion of bulk BP (Fig. 4b in the main article) is calculated by first relaxing the atomic coordinates to achieve forces less than 0.001 eV Å^{-1}, using the optimized lattice constants provided in the main article (a = 4.57 Å, b = 3.30 Å and c = 11.33 Å). Our lattice constants, taken from ref. 23, are consistent with other calculated values for monolayer and bilayer BP[24,25], and are reasonably close to the experimental lattice constants[26] (a = 4.38 Å, b = 3.31 Å and c = 10.48 Å). We perform density functional theory (DFT) simulations using GGA-PBE for exchange-correlation potential and PAW method to capture the effect of the atomic core, as implemented in VASP[27,28]. We employ a plane-wave

energy cutoff of 450 eV and a 11×9×9 Monkhorst-Pack-generated reciprocal-space grid (lattice vectors: a_1 = [a 0 0]; a_2 = [0 b/2 –c/2]; a_3 = [0 b/2 c/2]). Supplementary Figure 5 shows our calculated electronic dispersion of bulk BP, which is consistent with that reported in ref. 23, including a direct band gap of 0.15 eV. The dynamical matrix is constructed using the force constants extracted from the finite-displacement method (displacement of 0.01 Å, 3×5×5 supercell of the primitive cell with first number corresponding to a-axis), and then solved to obtain the phonon energies using Phonopy[29]. We used the phonon energies, calculated for a rectangular cell (a×b×c) with a 85×117×35 k-point grid in reciprocal space, to extract the number of modes per cross-sectional-area M_{ph} using LanTraP[30]. Although our computational approach is the same as that in ref. 23 (from which we take the lattice constants), our own total-energy minimization yields slightly different optimal lattice constants a = 4.56 Å, b = 3.31 Å and c = 11.32 Å. Supplementary Figure 6 compares the phonon dispersion of bulk BP using the lattice constants reported in ref. 23 and those obtained through our optimization. No significant differences in phonon energy are observed, thus we can safely use the lattice constants provided in ref. 23.

The phonon mean-free-path (MFP) for backscattering includes two contributions: *i)* phonon-phonon (Umklapp) scattering and *ii)* surface scattering of phonons on the finite thickness of the BP films. The intrinsic (bulk) MFP is written as[19]

$$\lambda_{\text{bulk}}\left(E,T\right)=2\left(\frac{\int_{\text{BZ}}v_{\parallel}^{+}\left(E,k_{\perp}\right)M_{\text{ph}}\left(E,k_{\perp}\right)\,\mathrm{d}k_{\perp}}{\int_{\text{BZ}}M_{\text{ph}}\left(E,k_{\perp}\right)\,\mathrm{d}k_{\perp}}\right)\tau_{\text{U}}\left(E,T\right), \tag{6}$$

where $v_{\parallel}^{+}$ is the band velocity along the transport direction for the forward moving states, $M_{\text{ph}}\left(E\right)=\int_{\text{BZ}}M_{\text{ph}}\left(E,k_{\perp}\right)\mathrm{d}k_{\perp}$, and $\tau_{\text{U}}\left(E,T\right)$ is intrinsic scattering time for Umklapp phonon-phonon scattering. The term in the parentheses depends only on phonon dispersion. Thus, the only missing information to calculate the thermal conductivity is the scattering time. We use the following phenomenological model of Umklapp scattering[21]

$$\tau_{\text{U}}^{-1}\left(E,T\right)=B\,E^{2}T\exp\left(-C/T\right)/\hbar^{2}, \tag{7}$$

where B and C are adjustable parameters. We choose C = 0 since we focus only on room temperature calculations and wish to eliminate one adjustable parameter. The film MFP is obtained by including surface scattering using the Fuchs-Sondheimer approach[19,20]

$$\lambda_{\text{film}}(E) = \lambda_{\text{bulk}}(E)\left[1 - \frac{3(1-p)}{2\delta}\int_1^{\infty}\left(\frac{1}{x^3} - \frac{1}{x^5}\right)\frac{1-\exp(-\delta x)}{1-p\exp(-\delta x)}\mathrm{d}x\right], \tag{8}$$

where $\delta = (4/3)(t/\lambda_{\text{bulk}})$, t is the BP film thickness and p is the specularity parameter controlling the degree of resistive scattering at the surface, with $p = 0$ and 1 corresponding to completely diffuse and specular scattering, respectively. To summarize, we adjust the two parameters B (Umklapp scattering) and p (surface scattering) to achieve the best agreement with the measured thermal conductivities (note that these parameters do not change depending on the transport direction, zigzag versus armchair). For $p < 1$, it can be shown that as the film thickness t is reduced, λ_{film} tends to be more isotropic along armchair and zigzag directions due to the increasing effect of surface scattering over the intrinsic Umklapp scattering.

With the number of modes per cross-sectional-area M_{ph} and the phonon MFP λ_{ph} determined, we were able to calculate the thermal conductivity of BP films and found good agreement with the experimental data using $B = 2 \times 10^{-19}$ s K^{-1} and $p = 0$ (completely diffusive surface scattering).

Although BP has anisotropic thermal transport characteristics, here we adopted a well-established and physics-based model for an isotropic scattering time distribution due to Umklapp scattering, which describes the temperature and energy dependence. This model was found to provide good agreement with experiment (see for example ref. 21). Since we are unaware of well-proven phenomenological models for Umklapp scattering time distributions that are anisotropic, we began by trying a widely-used and successful isotropic scattering model. Our results show that an isotropic scattering time distribution adequately reproduces the experimental thermal conductivity; there is no clear signature in the experimental data of an anisotropic Umklapp scattering rate (i.e. the observed anisotropy in the thermal conductivity is mostly the result of anisotropy in the modes). It is possible that any anisotropy in the Umklapp scattering time is not clearly perceived in the experimental data since surface scattering tends to make the MFP distribution more isotropic (i.e. long-MFP phonons are scattered more strongly than short-MFP phonons).

To examine what role the anisotropic MFP distribution (i.e. using an isotropic scattering time distribution, as described above) plays in the observed anisotropy in thermal conductivity, we compute the thermal conductivity using an isotropic MFP distribution that corresponds to the

value averaged over both zigzag and armchair directions (i.e. angle-averaged MFP) at each energy. Supplementary Figure 8 compares the calculated thermal conductivity using both anisotropic and isotropic MFP distributions. The isotropic MFP still yields significant anisotropy in thermal conductivity, although less than using an anisotropic MFP. The anisotropy in thermal conductivity is mostly a direct consequence of the anisotropy in the number of phonon modes.

Supplementary References

1. Wu, J., Mao, N., Xie, L., Xu, H. & Zhang, J. Identifying the Crystalline Orientation of Black Phosphorus Using Angle-Resolved Polarized Raman Spectroscopy. *Angew. Chemie* **127,** 2396–2399 (2015).

2. Sugai, S., Ueda, T. & Murase, K. Pressure dependence of the lattice vibration in the orthorhombic and rhombohedral structures of black phosphorus. *J. Phys. Soc. Japan* **50,** 3356–3361 (1981).

3. Xia, F., Wang, H. & Jia, Y. Rediscovering black phosphorus as an anisotropic layered material for optoelectronics and electronics. *Nat. Commun.* **5,** 4458 (2014).

4. Riedner, R. J. *et al.* Anisotropic Thermal Expansion and Compressibility of Black Phosphorus. *AIP Conf. Proc.* **17,** 8–20 (1974).

5. Retajczyk, T. F. & Sinha, A. K. Elastic Stiffness and Thermal Expansion Coefficients of Various Refractory Silicides and Silicon Nitride Films. *Thin Solid Films* **70,** 241–247 (1980).

6. Fei, R. & Yang, L. Lattice vibrational modes and Raman scattering spectra of strained phosphorene. *Appl. Phys. Lett.* **105,** 083120 (2014).

7. Beechem, T., Graham, S., Kearney, S. P., Phinney, L. M. & Serrano, J. R. Invited Article: Simultaneous mapping of temperature and stress in microdevices using micro-Raman spectroscopy. *Rev. Sci. Instrum.* **78,** 061301 (2007).

8. Beechem, T., Yates, L. & Graham, S. Invited Review Article: Error and uncertainty in Raman thermal conductivity measurements. *Rev. Sci. Instrum.* **86,** 041101 (2015).

9. Incropera, F., Lavine, A. & DeWitt, D. *Fundamentals of heat and mass transfer*. *John Wiley & Sons* (2011).

10. Pop, E., Varshney, V. & Roy, A. K. a. K. Thermal properties of graphene: Fundamentals and applications. *MRS Bull.* **37,** 1273–1281 (2012).

11. Luo, T. & Chen, G. Nanoscale heat transfer – from computation to experiment. *Phys. Chem. Chem. Phys.* **15,** 3389–3412 (2013).

12. Zink, B. L. & Hellman, F. Specific heat and thermal conductivity of low-stress amorphous Si–N membranes. *Solid State Commun.* **129,** 199–204 (2004).

13. Jain, A. & Goodson, K. E. Measurement of the Thermal Conductivity and Heat Capacity of Freestanding Shape Memory Thin Films Using the 3ω Method. *J. Heat Transfer* **130,** 102402 (2008).

14. Zhang, X. & Grigoropoulos, C. P. Thermal conductivity and diffusivity of free-standing silicon nitride thin films. *Rev. Sci. Instrum.* **66,** 1115 (1995).

15. Luo, Z. *et al.* Measurement of In-Plane Thermal Conductivity of Ultrathin Films Using Micro-Raman Spectroscopy. *Nanoscale Microscale Thermophys. Eng.* **18,** 183–193 (2014).

16. Bai, S., Tang, Z., Huang, Z. & Yu, J. Thermal Characterization of Thin Films Using Transient Thermoreflectance Technique. *IEEE Trans. Ind. Electron.* **56,** 3238–3243 (2009).

17. Mastrangelo, C. H., Tai, Y.-C. & Muller, R. S. Thermophysical properties of low-residual stress, Silicon-rich, LPCVD silicon nitride films. *Sensors Actuators A Phys.* **23,** 856–860 (1990).

18. Queen, D. R. & Hellman, F. Thin film nanocalorimeter for heat capacity measurements of 30 nm films. *Rev. Sci. Instrum.* **80,** 063901 (2009).

19. Pettes, M. T., Maassen, J., Jo, I., Lundstrom, M. S. & Shi, L. Effects of surface band bending and scattering on thermoelectric transport in suspended bismuth telluride nanoplates. *Nano Lett.* **13,** 5316–5322 (2013).

20. Jeong, C., Datta, S. & Lundstrom, M. Thermal conductivity of bulk and thin-film silicon: A Landauer approach. *J. Appl. Phys.* **111,** 093708 (2012).

21. Jeong, C., Datta, S. & Lundstrom, M. Full dispersion versus Debye model evaluation of lattice thermal conductivity with a Landauer approach. *J. Appl. Phys.* **109,** 073718 (2011).

22. Jeong, C., Kim, R., Luisier, M., Datta, S. & Lundstrom, M. On Landauer versus Boltzmann and full band versus effective mass evaluation of thermoelectric transport coefficients. *J. Appl. Phys.* **107,** 023707 (2010).

23. Qiao, J., Kong, X., Hu, Z.-X., Yang, F. & Ji, W. High-mobility transport anisotropy and linear dichroism in few-layer black phosphorus. *Nat. Commun.* **5,** 4475 (2014).

24. Qin, G. *et al.* Anisotropic intrinsic lattice thermal conductivity of phosphorene from first principles. *Phys. Chem. Chem. Phys.* **17,** 4854–4858 (2015).

25. Fei, R. & Yang, L. Strain-engineering the anisotropic electrical conductance of few-layer black phosphorus. *Nano Lett.* **14,** 2884–2889 (2014).

26. Brown, A. & Rundqvist, S. Refinement of the crystal structure of black phosphorus. *Acta Crystallogr.* **19,** 684–685 (1965).

27. Kresse, G. & Furthmüller, J. Efficient iterative schemes for ab initio total-energy calculations using a plane-wave basis set. *Phys. Rev. B* **54,** 11169–11186 (1996).

28. Kresse, G. & Furthmüller, J. Efficiency of ab-initio total energy calculations for metals and semiconductors using a plane-wave basis set. *Comput. Mater. Sci.* **6,** 15–50 (1996).

29. Togo, A., Oba, F. & Tanaka, I. First-principles calculations of the ferroelastic transition between rutile-type and CaCl2-type SiO2 at high pressures. *Phys. Rev. B* **78,** 134106 (2008).

30. Conrad, K., Maassen, J. & Lundstrom, M. LanTraP. https://nanohub.org/resources/lantrap. doi:10.4231/D3NP1WJ64 (2014).

31. Maassen, J. & Lundstrom, M. A computational study of the thermoelectric performance of ultrathin Bi2Te3 films. *Appl. Phys. Lett.* **102,** 093103 (2013).
